# Association between maternal exposure to particulate matter (PM_2.5_) and adverse pregnancy outcomes in Lima, Peru

**DOI:** 10.1038/s41370-020-0223-5

**Published:** 2020-04-30

**Authors:** V. L. Tapia, B. V. Vasquez, B. Vu, Y. Liu, K. Steenland, G. F. Gonzales

**Affiliations:** 1 Laboratorio de Reproducción y Endocrinología, LID, Universidad Peruana Cayetano Heredia, Lima, Peru; 2 Department of Biological and Physiological Sciences, Faculty of Sciences and Philosophy, Universidad Peruana Cayetano Heredia, Lima, Peru; 3 Environmental Health, Rollins School of Public Health, Emory University, Atlanta, GA USA

**Keywords:** Air pollution, Birth weight, PM_2.5_, Low birth weight, Fetal growth restriction, Small for gestational age, Preterm births

## Abstract

The literature shows associations between maternal exposures to PM_2.5_ and adverse pregnancy outcomes. There are few data from Latin America. We have examined PM_2.5_ and pregnancy outcomes in Lima. The study included 123,034 births from 2012 to 2016, at three public hospitals. We used estimated daily PM_2.5_ from a newly created model developed using ground measurements, satellite data, and a chemical transport model. Exposure was assigned based on district of residence (*n* = 39). Linear and logistic regression analyzes were used to estimate the associations between air pollution exposure and pregnancy outcomes. Increased exposure to PM_2.5_ during the entire pregnancy and in the first trimester was inversely associated with birth weight. We found a decrease of 8.13 g (−14.0; −1.84) overall and 18.6 g (−24.4, −12.8) in the first trimester, for an interquartile range (IQR) increase (9.2 µg/m^3^) in PM_2.5_. PM_2.5_ exposure was positively associated with low birth weight at term (TLBW) during entire pregnancy (OR: 1.11; 95% CI: 1.03–1.20), and at the first (OR: 1.11; 95% CI: 1.03–1.20), second (OR: 1.09; 95% CI: 1.01–1.17), and third trimester (OR: 1.10; 95% CI: 1.02–1.18) per IQR (9.2 µg/m^3^) increase. Higher exposure to PM_2.5_ was also associated with increased risk of small for gestational age (SGA). There were no statistically significant associations between PM_2.5_ exposure and preterm births (PTB). Exposure to higher concentrations of PM_2.5_ in Lima may decrease birth weight and increase the frequency of TLBW and SGA. Our study was inconsistent with the literature in finding no associations with preterm birth.

## Introduction

Air pollution is a major environmental concern worldwide. There is a large literature regarding particulate matter (PM) in air and its relationship with human health [1–3].

The metropolitan area of Lima, a megacity located in Peru, has been considered among the most contaminated cities in Latin America [4] with annual concentrations of PM_2.5_ ranging from 35 µg/m^3^ (Ate station) to 16 µg/m^3^ (Campo de Marte station). In 2015, it reached a mean value of 26 µg/m^3^ for 2015 [5]. These values are above that recommended by WHO (<10 µg/m^3^).

PM_2.5_ is a heterogeneous mixture of fine PM that can induce systemic inflammation, oxidative stress, and hemodynamic changes. In pregnant women, the placenta can be affected by PM reducing the blood flow, and delivering less oxygen and nutrients to the fetus [6]. Studies in animal models have demonstrated that exposure to fine PM can induce an inflammatory reaction in the fetal portion of the placenta [7]. This inflammatory reaction associated to elevated platelet count, IL-6 and peripheral blood mononuclear cell levels may alter the placental transport capacity [8].

Low birth weight at term (TLBW) (<2500 g) has been considered an important risk factor for noncommunicable diseases later in life such as coronary heart disease, stroke, type II diabetes, and hypertension [4]. Preterm birth (PTB) is the most common direct cause of neonatal morbidity and mortality. The PTB rate in 2015 was 7% in Peru [9] and 6.5% in Lima, respectively [10].

Previous meta-analyses have shown an association between high PM_2.5_ and TLBW, with odds ratios (ORs) ranging from 1.02 to 1.13 per 10 µg/m^3^ or interquartile range (IQR) increase, and with PTB, with ORs ranging from 1.02 to 1.14 [11–14]. These studies indicated that birth weight decreased 10–16 g per 10 µg/m^3^ increase in PM_2.5_.

Most studies about PM_2.5_ come from developed countries, with very few from Latin America. Lima has heterogeneous levels of PM_2.5_, offering large exposure contrasts for pregnant women living in different parts of Lima. To our knowledge there are no studies related to the effect of outdoor PM air pollution on pregnancy outcomes in Lima.

Our hypothesis was that higher PM_2.5_ has an association with lower birth weight, and would increase the risk of PTB, TLBW, and births that are small for gestational age (SGA). Therefore, the aim of the present study was to investigate the association between maternal exposure to PM_2.5_ overall during pregnancy, and at each trimester, in relation to adverse pregnancy outcomes in Lima, Peru, from 2012 to 2016.

## Material and methods

### Study area

Lima is located in the central coast of Peru at 150 m above sea level and has an approximate population of 9,320,000 inhabitants [15]. The average annual temperature is between 19 and 20 °C, with an average maximum temperature of 25 °C and a minimum of 19 °C.

**Fig. 1:**
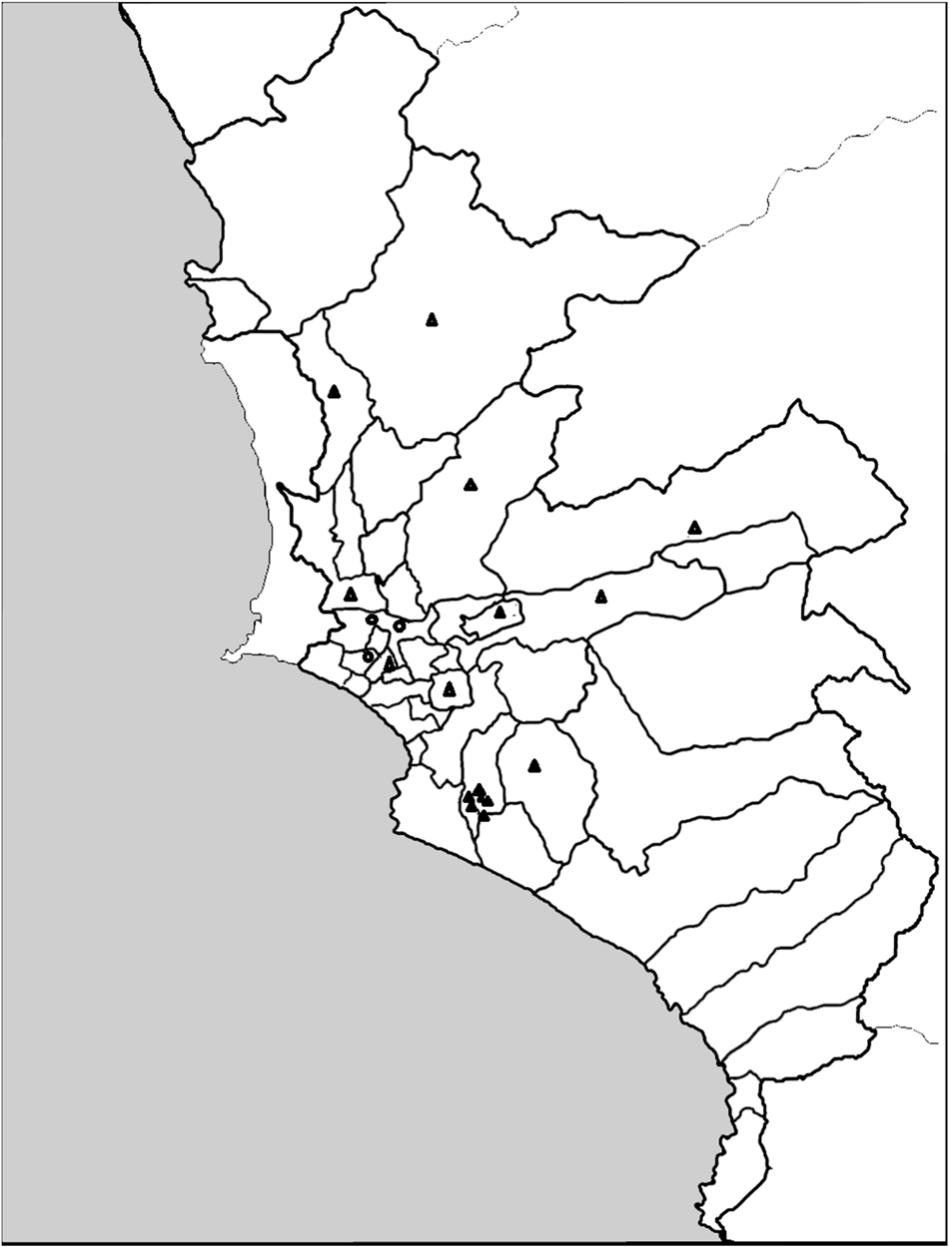
Map of Lima showing boundaries of districts and locations of monitoring stations and hospitals. Triangles are monitoring stations, circles are hospitals.

The metropolitan area of Lima is made up of 43 districts and divided into 4 zones: North Lima, Central Lima, East Lima, and South Lima. For the present study, we have assigned exposure based on the residential districts (*n* = 39) of pregnant women who delivered in three large public hospitals: National Maternal Perinatal Institute, (INMP in Spanish) and San Bartolome National Hospital (specializing in maternal and perinatal care), and Santa Rosa hospital, all located in downtown Lima (Fig. 1). We excluded four districts at high altitude (all above 570 m average altitude) due to uncertainty about the PM_2.5_ model predictions in these districts. The uncertainty was largely driven by the fact that ground-monitoring stations providing inputs to the PM_2.5_ model were all located below 375 m, requiring a large extrapolation to these four high districts, using the model’s prediction of the altitude effect. These districts (district numbers 150106, 150107, 150109, and 150118) represented only 4% of the total population of Lima, and hence their exclusion could have little bearing on the results.

### Study population

Birth records for the hospital were obtained from the three public hospitals for the years 2012–2016. These hospitals attend pregnant women from all over Lima. Two of them were exclusive maternal care facilities, and, together they attend 26% of the deliveries in Lima [15]. From 138,834 recorded births between January 2012 and December 2016, the following cases were excluded: multiple births (*n* = 7430), mothers residing outside the metropolitan area of Lima (*n* = 466), malformations (*n* = 855), stillbirths (*n* = 1469), and outlier data where birth weight that was greater or less than three standard deviations from the mean corresponding to gestational age (*n* = 340), missing residence district (*n* = 150), duplicate records (*n* = 1941), missing mother’s age (*n* = 69), and days without PM_2.5_ estimates (*n* = 3080), due to missing satellite date on some days. The remaining data for live newborns from singleton births coming from women aged 15–49 years old and living in the Metropolitan area of Lima, numbered a total of 123,034 births.

### Outcome variables

Birth weight (g) was defined as the first weight of the newborn measured in a calibrated scale by a trained hospital staff. PTB was defined by a delivery below 37 weeks of gestation. TLBW was defined as birth weight less than 2500 g at 37–42 weeks of gestation. SGA was defined as the 10th percentile of weight by gestational age. Cutoff weights to determined SGA were developed using the birth weight-*Z*-score adjusted for gestational age, determined from INTERGROWTH tables (https://intergrowth21.tghn.org/).

### Outdoor PM_2.5_ data

Some ground-monitoring PM_2.5_ data in Lima were available from March 2010 through December 2016, from monitors in ten stations located in the four zones of Lima and belonging to the Peruvian National Service of Meteorology and Hydrology (SENAMHI, in Spanish, an agency of the Ministry of the Environment), and monitors in six stations in southern Lima operated from 2011 to 2012 by Johns Hopkins University [16] (Fig. 1). However, ground measurements were not consistently available on a daily basis, covering only about 10% of days in the study period. Thus, we based our PM_2.5_ exposure data from a model developed by Vu et al. [17], Briefly Vu et al. used satellite measurement and chemical transport model data to predict PM_2.5_ in Lima. The resulting model predicted daily PM_2.5_ in Lima at a 1 km^2^ spatial resolution and fits the observed data well, with an *R*-square of 0.70 (mean difference between ground and predicted measurements 0.09 µg/m^3^). The full methodology employed in this study can be finding in [17] as well.

Our birth records provided data on district of residence but not exact address. Hence we assigned to each mother the population-weighted average PM_2.5_ across her district for each day she was in the study. We then calculated several exposure variables for each birth: (1) average exposure to PM_2.5_ during the entire pregnancy defined as the average of pollutant concentration from week 1 to birth, and (2) average exposure by trimester of pregnancy, defined as the average of PM_2.5_ in the first (1–12 weeks), second (13–26 weeks), and the third (week 27 to birth) trimester of pregnancy.

### Gestational age and covariates

Gestational age at delivery was determined by the last menstrual period. For this variable, 105 values were missing in the database and were replaced by the gestational age determined by physical examination according to the Capurro methodology [18].

The following characteristics from mothers were also obtained from the database: mother’s age at delivery (categorized as <22 years, 22–39 years and >39 years), body mass index (BMI) according to WHO (low: <22; normal: 22–24.9; overweight: 25–29.9; obesity: ≥ 30 Kg/m^2^), and parity (0/1, >1). BMI was missing from half of the database and we generally did not include it in our models, but when we did, the results for PM_2.5_ were generally similar to those omitted. Variables for prenatal care and the presence of preeclampsia during pregnancy were included in the analysis as dichotomous variables (yes/no).

Prenatal care visits was defined as the number of antenatal visits to the health facility made throughout pregnancy. It was dichotomized as 0 or 1 visit, vs >1 visits.

Preeclampsia was defined as the presence of pregnancy-induced hypertension (arterial systolic pressure of ≥140 mm Hg and/or arterial diastolic pressure of ≥90 mm Hg) and proteinuria (≥300 mg/24 h) after 20 weeks of gestation in otherwise normotensive women.

Percentage of poverty was defined as the percentage of households within a district with a self-reported family income below what is needed for sustenance, food, clothing, or shelter by the Peruvian Census [15]. We dichotomized poverty into districts above and below the median poverty level.

Smoking was not considered as the rate among pregnant women in Peru is very low (<1%) and data were not consistently available [19]. All the variables described above where chosen based on a priori considerations of possible confounders. We included year in preliminary models but it did not improve model fit and did not change exposure estimates, so it was not included in final models.

We did not include hospital or district as either fixed or random effects because our exposure was assigned based on district of residence, and spatial contrast across districts was our primary exposure contrast, such that inclusion of either hospitals (which served primarily certain districts), or district itself, limited our ability to detect exposure contracts. We also had a number of individual-level potential confounders, limiting likely confounding by district.

A study in the National Maternal Perinatal Institute in Lima showed that ~16% of pregnant women at that hospital were diagnosed with gestational diabetes mellitus (GDM); in that study GDM was diagnosed using an Oral Glucose Tolerance Test performed between 24 and 28 gestational weeks [20]. However, such testing is not routinely done, and we lacked data on this potential confounder. GDM is associated with higher birth weight [21, 22]. GDM has also been linked to lower SES [23], which is associated with higher air pollution in our data (mean in districts below the midpoint poverty index 19.1 μg/m^3^, mean in districts above the midpoint, 22.8 μg/m^3^). Hence as a confounder GDM would be expected to bias our results to the null, making our estimates conservative.

### Statistical analyses

Data were collected in an Excel spreadsheet and analyzed using SAS version 9.4 (SAS Institute Inc., Cary, NC, USA). Generalized linear models were used to explore the relationship between PM_2.5_ exposure and birth weight as a continuous variable. Logistic regression analysis was used to determine the relationship between PM_2.5_ exposure during pregnancy with TLBW, SGA, and PTB. Covariates which by themselves attained a *p* value < 0.1 were included one by one in the models, and those that retained statistical significance at *p* < 0.05 were included in the final model. Maternal age, parity, prenatal care, gestational age, poverty, and preeclampsia were included in all final models, with the exception of poverty, which was not included in the model for TLBW.

In addition to continuous PM_2.5_, exposure was also categorized into quartiles (based on the data over the entire study period): Q1st: 12.74–16.83 µg/m^3^; Q2nd: 16.84–18.42 µg/m^3^: Q3rd: 18.43–26.04 µg/m^3^, and Q4th: 26.05–41.6 µg/m^3^, Exposure variables were also generated for each trimester of pregnancy. Regression models were run for exposure during the entire pregnancy and also by trimester of pregnancy. By way of reference, WHO recommends PM_2.5_ concentration below 10 µg/m^3^ as an annual mean, while the Peruvian Ministry of the Environmental in Peru recommends 25 µg/m^3^.

The protocol was approved by Ethics Review Committee of Cayetano Heredia University (SIDISI code 101546).

The computer code to conduct our analyses is available on request to the authors.

## Results

From the overall population studied (123,034 births), among those with full-term births, 2074 (1.8%) were TLBW, and from the overall population, 8897 (7.2%) were PTB (Table 1). In the full-term birth group, those with TLBW were more likely to be younger, without prenatal care, and have preeclampsia.

**Table 1:** Demographics and obstetric characteristic according to adverse outcome in Lima city, 2012–2016.

Characteristic	TLBW (2074)	PTB (8897)	Total (112063)
Age (years)			
<22	548 (26.4)	2010 (22.6)	27,771 (24.8)
22–39	1372 (66.1)	6265 (70.4)	79,234 (70.7)
>39	154 (7.4)	620 (7.0)	5030 (4.5)
Missing	–	2 (0.00)	28 (0.001)
Parity			
>1	1640 (74.9)	6468 (70.4)	65,190 (69.1)
0–1	424 (24.6)	2250 (27.6)	32,365 (28.8)
Missing	10 (0.5)	179 (2.0)	2568 (2.1)
Preeclampsia (%)			
No	1818 (87.7)	7596 (85.4)	108,399 (96.7)
Si	256 (12.3)	1301 (14.6)	3664 (3.3)
Poverty (%)			
No	1068 (51.5)	4541 (51.0)	56,181 (50.1)
Si	1006 (48.5)	4356 (48.9)	55,882 (49.9)
Prenatal care (%)			
Without control	526 (26.2)	2487 (29.0)	21,158 (18.9)
Control	1479 (70.5)	4089 (67.2)	88,345 (78.8)
Missing	69 (3.3)	333 (3.8)	2560 (2.3)
Birth weight (g)	2224.0 ± 316.4	2280.5 ± 3872	3356 ± 420
Gestational age (weeks)	38.3 ± 1.1	34.0 ± 2.69	39.2 ± 0.98

*TLBW* low birth weight at term (<2500 g birth weight at term), *PTB* preterm births (<37 weeks of gestational age).

For the mothers in our study, in the residential district PM_2.5_ concentration across all days ranged between 12.7 µg/m^3^ and 41.6 µg/m^3^, showing a mean value of 22.3 µg/m^3^ (SD: 5.43) and median 21.8 µg/m^3^, and an IQR of 9.2 µg/m^3^. The highest concentrations of PM_2.5_ were observed in the eastern zone of Lima, and the lowest concentrations in the center zone of Lima (Table 2).

**Table 2:** Average concentrations of PM_2.5_ (µg/m^3^) according to district in Lima, 2012–2016^a^.

No.	District	Mean PM_2.5_ (µg/m^3^)	SD	No.	District	Mean PM_2.5_ (µg/m^3^)	SD
North Lima 21.3							
1	Ancon	19.8	0.91	5	Puente Piedra	24.7	1.08
2	Comas	24.6	1.05	6	San Martin de Porras	16.6	0.59
3	Independencia	20.9	0.76	7	Santa Rosa	19.1	0.65
4	Los Olivos	17.4	0.68				
Central Lima 16.4							
8	Cercado de Lima	16.7	0.60	16	Miraflores	15.4	0.55
9	Barranco	15.4	0.48	17	Rimac	18.4	0.79
10	Breña	16.1	0.54	18	San Borja	17.7	0.67
11	Jesus María	15.1	0.61	19	San Isidro	15.8	0.53
12	La Victoria	17.4	0.66	20	San Luis	18.4	0.71
13	Lince	15.8	0.59	21	San Miguel	15.7	0.42
14	Magdalena	15.0	0.47	22	Santiago de Surco	18.4	0.56
15	Pueblo Libre	15.4	0.48	23	Surquillo	15.9	0.55
South Lima 19.1							
24	Chorrillos	16.3	0.39	30	San Bartolo	18.9	2.13
25	Lurin	16.9	0.37	31	San Juan de Miraflores	18.3	0.55
26	Pachacamac	24.9	0.69	32	Santa Maria	16.3	0.48
27	Pucusana	16.2	0.37	33	Villa El Salvador	17.6	0.51
28	Punta Hermosa	17.3	0.85	34	Villa Maria del Triunfo	22.3	0.71
29	Punta Negra	17.2	0.84				
East Lima 26.2							
35	Ate	26.3	1.48	38	San Juan de Lurigancho	28.4	1.65
36	El Agustino	24.4	1.35	39	Santa Anita	25.7	1.76
37	La Molina	26.3	1.09				

North Lima represents 7 districts; Central Lima has 16 districts, South Lima includes 11 districts and East Lima, three districts.

*SD* standard deviation.

^a^PM_2.5_ concentrations comes from the modeled data [18].

Table 3 shows the results of the regression analysis between birth weight and maternal PM_2.5_ exposure. Significant associations were observed with PM_2.5_ (continuous) exposure during the entire pregnancy, and during the first trimester, but not during the second and third trimester. An IQR (9.2 µg/m^3^) increase in PM_2.5_ concentrations during the entire pregnancy was associated with 8.13 g decrease in birth weight (−14.0, −1.84 g, *R*^2^ 0.39; *p* < 0.0001). We also found a decrease of 18.6 g (95% CI −24.4, −12.3 g) per IQR (9.2 µg/m^3^) increase in PM_2.5_ during the first trimester of pregnancy. In this trimester, the highest quartile of PM_2.5_ exposure (>25.3 µg/m^3^) was associated with lowest birth weight (−26.8 g; 95% CI −36.9, −17.8). We found the similar results when we restricted the data to full-term births. There was a decrease in birth weight largely restricted to PM_2.5_ in the first trimester.

**Table 3:** Regression models for birth weight and PM_2.5_ exposure at different times of pregnancy.

Variable	Change in birth weight (g) per IQR PM_2.5_
PM_2.5_ entire pregnancy (µg/m^3^)	**−8.13** (−14.0 −1.84)
PM_2.5_ entire pregnancy	
Q1st (12.7–16.83)	0
Q2nd (16.84–18.42)	12.6 (5.07 19.7)
Q3rd (18.43–26.04)	6.42 (−3.15 14.9)
Q4th (26.05–41.6)	−3.51 (−13.5 6.49)
PM_2.5_ first trimester (µg/m^3^)	**−18.6** (−24.4 −12.8)
PM_2.5_ first trimester	
Q1st (12.7–16.83)	0
Q2nd (16.84–18.42)	−3.70 (−11.6 2.64)
Q3rd (18.43–26.04)	−4.64 (−13.9 3.38)
Q4th (26.05–41.6)	**−26.8** (−36.9 −17.8)
PM_2.5_ second trimester (µg/m^3^)	0.01 (−5.52 5.245)
PM_2.5_ second trimester	
Q1st (12.7–16.83)	1.0
Q2nd (16.84–18.42)	11.2 (3.45 18.0)
Q3rd (18.43–26.04)	3.76 (−3.97 13.4)
Q4th (26.05–41.6)	4.22 (−9.21 13.6)
PM_2.5_ third trimester (µg/m^3^)	−2.68 (−7.92 2.55)
PM_2.5_ third trimester	
Q1st (12.7–16.83)	1.0
Q2nd (16.84–18.42)	8.05 (0.84 15.2)
Q3rd (18.43–26.04)	−1.22 (−9.75 7.29)
Q4th (26.05–41.6)	−1.48 (−10.7 7.83)

Referent category. Per IQR of PM_2.5_ (9.2 µg/m^3^). Models adjusted for maternal age, parity, preeclampsia, gestational age, prenatal care, and poverty.

Bold values indicate statistical significance *p* < 0.05.

The results of the logistic regression analysis to assess the relationship between PM_2.5_ and TLBW are shown in Table 4. There was a significant increased risk of TLBW per IQR (9.2 µg/m^3^) increase in PM_2.5_ concentration observed during the entire pregnancy (OR 1.11; 95% CI: 1.03–1.20) and all trimesters (OR: 1.11; 95% CI 1.03–1.20; OR 1.09, 95% CI 1.01–1.17; OR: 1.01, 95% CI 1.00–1.03; respectively) after controlling for age, parity, gestational age, preeclampsia, and prenatal care. In quartile analyses the highest risk was found in the fourth quartile of PM_2.5_.

**Table 4:** Associations between low birth weight at term and exposure of PM_2.5_ during the pregnancy, 2012–2016.

Variable	Risk of TLBW per IQR PM_2.5_
PM_2.5_ entire pregnancy (µg/m^3^)	**1.11** (1.03 1.20)
PM_2.5_ entire pregnancy	
Q1st (12.7–16.83)	1.0
Q2nd (16.84–18.42)	0.93 (0.81 1.05)
Q3rd (18.43–26.04)	0.99 (0.83 1.07)
Q4th (26.05–41.6)	**1.09** (1.01 1.18)
PM_2.5_ first trimester (µg/m^3^)	**1.11** (1.03 1.20)
PM_2.5_ first trimester	
Q1st (12.7–16.83)	1.0
Q2nd (16.84–18.42)	0.94 (0.86 1.02)
Q3rd (18.43–26.04)	0.94 (0.83 1.07)
Q4th (26.05–41.6)	**1.14** (1.05 1.23)
PM_2.5_ second trimester (µg/m^3^)	**1.09** (1.01 1.17)
PM_2.5_ second trimester	
Q1st (12.7–16.83)	1.0
Q2nd (16.84–18.42)	0.98 (0.91 1.18)
Q3rd (18.43–26.04)	1.02 (0.95 1.23)
Q4th (26.05–41.6)	1.05 (0.98 1.27)
PM_2.5_ third trimester (µg/m^3^)	**1.10** (1.02 1.18)
PM_2.5_ third trimester	
Q1st (12.7–16.83)	1.0
Q2nd (16.84–18.42)	0.96 (0.88 1.14)
Q3rd (18.43–26.04)	0.98 (0.89 1.15)
Q4th (26.05–41.6)	**1.09** (1.01 1.18)

Per IQR of PM_2.5_ (9.21 µg/m^3^). Model adjusted for maternal age, parity, preeclampsia, gestational age, and prenatal care.

Bold values indicate statistical significance *p* < 0.05.

The results of the logistic regression analysis to assess the relation between PM_2.5_ and SGA are shown in Table 5. We found significant increased risk of SGA per IQR (9.2 µg/m^3^) increase in PM_2.5_ concentrations over the entire pregnancy (OR 1.04; 95% CI: 1.01–1.08), during the first (OR 1.07; 95% CI 1.03–1.10) and third trimester (OR 1.04; 95% CI 1.00–1.07), after controlling for age, parity, preeclampsia, and prenatal care. Categorical analyses found the highest risk concentrated in the highest quartile of PM_2.5_ exposure overall and in the first and third trimester.

**Table 5:** Associations between small for gestational age and exposure of PM_2.5_ during the pregnancy, 2012–2016.

Variable	Odds ratio for SGA per IQR PM_2.5_
PM_2.5_ entire pregnancy (µg/m^3^)	**1.04** (1.01 1.08)
PM_2.5_ entire pregnancy	
Q1st (12.7–16.83)	1.0
Q2nd (16.84–18.42)	0.59 (0.35 0.97)
Q3rd (18.43–26.04)	1.11 (0.67 1.82)
Q4th (26.05–41.6)	1.42 (0.86 2.34)
PM_2.5_ first trimester (µg/m^3^)	**1.07** (1.03 1.10)
PM_2.5_ first trimester	
Q1st (12.7–16.83)	1.0
Q2nd (16.84–18.42)	0.70 (0.60 1.16)
Q3rd (18.43–26.04)	1.12 (0.68 1.85)
Q4th (26.05–41.6)	**2.16** (1.31 3.55)
PM_2.5_ second trimester (µg/m^3^)	1.02 (0.99 1.05)
PM_2.5_ second trimester	
Q1st (12.7–16.83)	1.0
Q2nd (16.84–18.42)	0.64 (0.38 1.06)
Q3rd (18.43–26.04)	1.53 (0.94 2.50)
Q4th (26.05–41.6)	1.01 (0.62 1.66)
PM_2.5_ third trimester (µg/m^3^)	**1.04** (1.00 1.07)
PM_2.5_ third trimester	
Q1st (12.7–16.83)	1.0
Q2nd (16.84–18.42)	0.99 (0.59 1.65)
Q3rd (18.43–26.04)	1.51 (0.91 2.52)
Q4th (26.05–41.6)	1.41 (0.85 2.36)

Per IQR of PM_2.5_ (9.21 µg/m^3^). Model adjusted for maternal age, parity, preeclampsia, and prenatal care.

Bold values indicate statistical significance *p* < 0.05.

No significant associations were observed between exposure to PM_2.5_ and preterm birth (Table 6).

**Table 6:** Associations between maternal exposure to PM_2.5_ and preterm births, 2012–2016.

Variable	Risk of preterm birth per IQR PM_2.5_
PM_2.5_ entire pregnancy	0.98 (0.95 1.02)
PM_2.5_ entire pregnancy (µg/m^3^)	
Q1st (12.7–16.83)	1.0
Q2nd (16.84–18.42)	0.87 (0.76 0.87)
Q3rd (18.43–26.04)	1.12 (0.98 1.11)
Q4th (26.05–41.6)	0.95 (0.83 0.94)
PM_2.5_ first trimester (µg/m^3^)	1.01 (0.95 1.07)
PM_2.5_ first trimester	
Q1st (12.7–16.83)	1.0
Q2nd (16.84–18.42)	0.94 (0.84 0.96)
Q3rd (18.43–26.04)	1.05 (0.93 1.05)
Q4th (26.05–41.6)	0.98 (0.89 1.01)
PM_2.5_ second trimester (µg/m^3^)	1.00 (0.97 1.04)
PM_2.5_ second trimester	
Q1st (12.7–16.83)	1.0
Q2nd (16.84–18.42)	0.99 (0.93 1.06)
Q3rd (18.43–26.04)	1.03 (0.97 1.11)
Q4th (26.05–41.6)	0.98 (0.93 1.06)
PM_2.5_ third trimester (µg/m^3^)	0.98 (0.94 1.01)
PM_2.5_ third trimester	
Q1st (12.7–16.83)	1.0
Q2nd (16.84–18.42)	1.01 (0.95 1.08)
Q3rd (18.43–26.04)	1.02 (0.96 1.09)
Q4th (26.05–41.6)	0.97 (0.91 1.03)

Per unit of PM_2.5_ (9.21 µg/m^3^). Model adjusted for parity, maternal age, prenatal care, gestational age, and preeclampsia.

We also explored possible differences between male and female births. We found some differences for birth weight, but little difference for TLBW or SGA. For girls, birth weight decreased 23.8 g (95% CI 15.9–31.8) per IQR (9.2 µg/m^3^) increase of PM_2.5_ in the first trimester while boys had a 12.6 g (95% CI 4.7–20.5) decrease. In girls, the OR of TLBW associated with an IQR increase in PM_2.5_ across all trimesters was 1.13 (95% CI 1.03–1.24), while boys had an OR of 1.09 (95% CI 1.00–1.19). For SGA, for an IQR increase in PM_2.5_ across all trimesters, girls had an OR of 1.05 (95% CI 1.00–1.10) while boys had an OR of 1.04 (95% CI 0.99–1.09). For PTB, there was virtually no effect of PM_2.5_ when analyses were conducted for each sex separately.

## Discussion

The present study aimed to determine the relationship between the exposures to outdoor PM_2.5_ and adverse pregnancy outcomes, in a city considered one of the more contaminated among large cities in Latin America. The PM_2.5_ concentration average for mothers in Lima as a whole was 22.3 µg/m^3^ (±5.43). Eastern areas in Lima had higher values, while Central Lima had the lowest. The variability observed between districts is due to the morphological and atmospheric characteristics that determine the air quality in Lima. Coastal winds heading south to north, the Andes Mountain on the east, and thermal inversions, cause the pollution loads to be deposited in the eastern and northeastern areas of Lima [5]. This variability between districts motivated our use of district-level PM_2.5_ as our exposure measure.

Our results showed that exposure to PM_2.5_ was associated with birth weight, SGA, and TLBW. We observed a decrease in birth weight with higher PM_2.5_ over the entire pregnancy and in the first trimester, while risks for TLBW and SGA were elevated in each of the three trimesters. This is an important finding since the mean value of PM_2.5_ concentration for our mothers observed in Lima (22.3 µg/m^3^) is considered by the Peruvian authorities as normal (recommended level for PM_2.5_ is <25 µg/m^3^), but resulted in higher risk of adverse pregnancy outcomes.

Three prior meta-analyses [12, 13, 24] found the higher PM_2.5_ was associated with higher risk of LBW or TLBW. Our results for TLBW are generally consistent with these reviews.

Regarding birth weight as a continuous variable, two meta-analysis [11, 13] found lower birth weight with higher PM_2.5_, during the entire pregnancy, and in particular in the third trimester [13]. Our results are generally consistent with these meta-analyses, although our effects were largely confined to the first trimester exposure.

However, not all studies have found the strongest effects on birth weight due to third trimester exposure. In one study by Kumar et al. [25], birth weight showed a stronger inverse association with PM_2.5_ exposure during the first trimester than during the second and third trimesters. Another study by Li et al. [26] had similar findings. A recent article by Blum et al. [27] exposing pregnant mice to PM_2.5_ found lower birth weight after exposure in both early and late gestational periods.

Oxidative stress produced by PM_2.5_ exposure during the first trimester could interfere with the normal development of the placenta, affecting placental mitochondria and, therefore, the ability of the placenta to support the growing fetus [28]. The fastest growth of the fetus occurs around 32 weeks of gestation, which is the age in which the fetus may be more vulnerable to deleterious stimulus.

The PM_2.5_ exposure during the first trimester may also show a stronger association with body weight than the exposure during the second and third trimester, because the fetus size is relatively small during early gestation period. Thus, the same PM_2.5_ exposure during the early stage of gestation period may translate to a high dose of PM_2.5_, given the smaller fetus size [25]. On the other hand, for TLBW and SGA, we found an effect of higher PM_2.5_ for exposure in both early and late in pregnancy.

With respect to PTB, three prior meta-analyses [12, 14, 29] found significant positive associations with PM_2.5_, while we did not. We have no explanation for this discrepancy. In our data, PM_2.5_ seemed to decrease fetal growth but not lead to prematurity. It may be that the specific nature of the PM_2.5_ in Lima, which derives primarily from vehicular emissions rather than industrial sources, is associated with fetal growth but not prematurity.

One strength of our study is the use of a predictive model that estimated daily PM_2.5_ concentrations for each district of Lima. This model allowed the construction of consistent long-term historical measurements in replacement of the sparse data available from ground monitors, and the assignment of PM_2.5_ to local residential areas. In addition, we used the perinatal records of three large specialized public hospital located in the center of Lima, which received pregnant women from all the districts of the city.

One limitation of our study is the lack of data on co-pollutants such as ozone, or NO_2_, as reported in other articles [30, 31]. Another limitation is our lack of data regarding the occupation of the women in the study (a potential confounder), as well as the location of the occupation, which will have led to some mismeasurement of estimated air pollution exposure for women who worked outside of their district. We also lack information of some other potential confounders such as alcohol consumption, but previous studies in Peru showed low consumption of alcohol by pregnant women [32, 33].

Identification of an association of air pollution with adverse pregnancy outcomes has substantial public health implications in Lima. We note that while WHO recommends an annual limit of 10 µg/m^3^ for PM_2.5_, the Peruvian Ministry of Environment recommends a much higher limit of 25 µg/m^3^.

## Conclusion

This is the first study that correlates the exposure to PM_2.5_ with pregnancy outcomes in Lima. The results supported previous findings that environmental PM_2.5_ exposure can be hazardous to pregnancy, regarding birth weight, low birth weight, and births SGA, but not for PTB, where we found no effect for PM_2.5_. We found our most significant association of PM_2.5_ with birth weight during the first trimester. Our study provides new evidence regarding the effects of PM_2.5_ on pregnancy outcomes, suggesting that the existing Peruvian standards are too high.
